# Patients’ and physicians’ experiences with remote consultations in primary care during the COVID-19 pandemic: a multi-method rapid review of the literature

**DOI:** 10.3399/BJGPO.2021.0192

**Published:** 2022-04-20

**Authors:** Pradipti Verma, Robert Kerrison

**Affiliations:** 1 Research Department of Behavioural Science and Health, University College London, London, UK; 2 School of Health Sciences, University of Surrey, Surrey, UK

**Keywords:** COVID-19, Nonverbal Communication, physician-patient relations, primary care, remote consultations, SARS-CoV-2, systematic review, telephone consultations, video consultations, virtual consultations

## Abstract

**Background:**

During the COVID-19 pandemic, many countries implemented remote consultations in primary care to protect patients and staff from infection.

**Aim:**

The aim of this review was to synthesise the literature exploring patients’ and physicians’ experiences with remote consultations in primary care during the pandemic, with the further aim of informing their future delivery.

**Design & setting:**

Rapid literature review.

**Method:**

PubMed and PsychInfo were searched for studies that explored patients’ and physicians’ experiences with remote consultations in primary care. To determine the eligibility of studies, their titles and abstracts were reviewed, before the full article. Qualitative and quantitative data were then extracted from those that were eligible, and the data synthesised using thematic and descriptive synthesis.

**Results:**

A total of 24 studies were eligible for inclusion in the review. Most were performed in the US (*n* = 6, 25%) or Europe (*n* = 7, 29%). Patient and physician experiences were categorised into perceived ‘advantages’ and ‘issues’. Key advantages experienced by patients and physicians included ‘reduced risk of COVID-19’ and ‘increased convenience’, while key issues included ‘a lack of confidence in or access to required technology’ and a ‘loss of non-verbal communication’ which degraded clinical decision-making.

**Conclusion:**

This review identified a number of advantages and issues experienced by patients and physicians using remote consultations in primary care. The results suggest that, while remote consultations are more convenient and protect patients and staff against COVID-19, they result in the loss of valuable non-verbal communication, and are not accessible to all.

## How this fits in

Previous studies have explored patients’ and primary care physicians’ (PCPs) experiences with remote consultations during the COVID-19 pandemic, but none have synthesised them in the form of a review. To collate what is known, this study extracted data from the quantitative, qualitative, and mixed-methods literature, and used thematic and narrative synthesis methods to pool and interpret the results. The findings of this study highlight that remote consultations are considered more convenient than face-to-face consultations, and protect patients and staff from COVID-19, they result in the loss of valuable non-verbal communication, and are not accessible to all. The results of this study can be used to inform the future delivery of remote consultations in primary care in a "post-COVID-19" world.

## Introduction

SARS-CoV-2 is a novel respiratory virus that causes the disease COVID-19, with an estimated infection case fatality rate of 0.15%.^1^ COVID-19 is not the first discovered coronavirus,^2^ but COVID-19 is of contemporary relevance owing to its ability to spread and cause severe disease; between Jan 1 2020 and December 31 2021, >5 million people were reported to have died globally from the disease (estimates suggest mortality could be three times higher), and the situation has been declared a ‘global pandemic’ by the World Health Organization.^3^

COVID-19 is transmitted through droplets emitted when sneezing, coughing, or speaking, and can enter the body through the eyes, nose, or mouth.^4^ To protect individuals, and the health services that treat them, several social distancing and disease prevention measures have been implemented by governments the world over, with a view to abolishing these once immunity through vaccination and improvements in treatment are developed.^5^

Among the measures implemented, face-to-face consultations for primary care appointments were discouraged in most countries.^6^ This has led to a radical change in the delivery of primary care, with visits increasingly being conducted via remote consultation.^6^ In the US, for example, 46% of patients used telephone consultations in 2020, which was considerably higher than 2019, when only 11% reported using telephone consultations.^7^ Similar patterns have been observed in the UK, where <50% of appointments were face-to-face in 2020 versus >80% in 2019,^8^ with the majority replaced by telehealth (although providers are also being encouraged to increase the number of video consultations they perform).^9,10^

While remote consultations have been beneficial in protecting patients and physicians from COVID-19 during the pandemic, there is uncertainty about their future use, as well as the extent to which patients and PCPs are satisfied with them. The aim of this review, therefore, was to synthesise data on patients’ and PCPs’ experiences with remote consultations in the primary care setting to inform future research and policy in this area.

## Method

### Search strategy and study design

Owing to resource restrictions (staff availability and funding), a rapid review of the literature was performed. Rather than running a single search with the full list of search terms, therefore, an initial search was performed (see Table 1) using a narrow selection of search terms (guided by the PICOS [Population, Intervention, Comparison, Outcome and Study design] framework; see Supplementary Table S1 for details). This was successively expanded by adding a small number of additional search terms (to each PICOS component), from a pool of search terms identified from previous literature, until only a very small number of newly identified articles were eligible for inclusion in the review.

**Table 1.** Results of successively expanding the search string until the time newly identified articles potentially eligible (on abstract review) was around 1% of the total number of articles found by the search

Search strings	Publications(duplicate removed)**, *n***	Publications selectedby two reviewerson title review, *n*	New publications selectedby two reviewerson abstract review, *n*	Publications eligible, %
**PubMed**				
**Search 1:** ((Patients OR Primary Care OR General Practice) AND (COVID OR COVID-19 OR Coronavirus OR SARS-COV-2 OR Pandemic) AND (Video Consult* OR Telephone consult* OR Online Consult*) AND (Barriers OR Facilitators OR Attitudes) AND (Mixed Methods OR Qualitative OR quantitative OR Survey OR Questionnaire OR Interview* OR Focus group* OR Multi Methods))	112	53	25	22.3%
**Search 2:** ((Patients OR Primary Care OR General Practice OR Family clinician) AND (COVID-19 OR COVID OR Coronavirus OR SARS-COV-2 OR Pandemic) AND (Video Consult* OR Telephone consult* OR Online Consult* OR Telehealth OR Telemedical OR Telecare OR Telemedicine) AND (Barriers OR Facilitators OR Attitudes OR Challenges) AND (Quantitative OR Qualitative OR Mixed Methods OR Survey OR Questionnaire OR Interview* OR Focus group* OR Multi Methods))	599	150	42	7.0%
**Search 3:** ((Patients OR Primary Care OR General Practice OR Family clinician OR Family Physician*) AND (COVID-19 OR COVID OR Coronavirus OR SARS-COV-2 OR Pandemic) AND (Video Consult* OR Telephone consult* OR Online Consult*OR Telehealth OR Telemedical OR Telecare OR Telemedicine OR Digital Health OR "mHealth" OR Connected Care) AND (Barriers OR Facilitators OR Attitudes OR Challenges OR Factors) AND (Quantitative OR Qualitative OR Mixed Methods OR Survey OR Questionnaire OR Interview* OR Focus group* OR Multi Methods))	879	213	21	2.4%
**Search 4:** ((Patients OR Primary Care OR General Practice OR Family clinician OR Family Physician* OR General Practitioner) AND (COVID-19 OR COVID OR Coronavirus OR SARS-COV-2 OR Pandemic) AND (Video Consult* OR Telephone consult* OR Online Consult*OR Telehealth OR Telemedical OR Telecare OR Telemedicine OR Digital Health OR "mHealth" OR Connected Care OR Virtual clinic OR Virtual Medicine OR Video visit) AND (Barriers OR Facilitators OR Attitudes OR Challenges OR Factors OR Perceptions) AND (Quantitative OR Qualitative OR Mixed Methods OR Survey OR Questionnaire OR Interview* OR Focus group* OR Multi Methods))	1058	236	12	1.1%
**PsyInfo**				
**Search 4:** ((Patients OR Primary Care OR General Practice OR Family clinician OR Family Physician* OR General Practitioner) AND (COVID-19 OR COVID OR Coronavirus OR SARS-COV-2 OR Pandemic) AND (Video Consult* OR Telephone consult* OR Online Consult*OR Telehealth OR Telemedical OR Telecare OR Telemedicine OR Digital Health OR "mHealth" OR Connected Care OR Virtual clinic OR Virtual Medicine OR Video visit) AND (Barriers OR Facilitators OR Attitudes OR Challenges OR Factors OR Perceptions) AND (Quantitative OR Qualitative OR Mixed Methods OR Survey OR Questionnaire OR Interview* OR Focus group* OR Multi Methods))	1061	237	0	0.0%

The exact combination and order in which search terms were added to the search string were determined by running multiple searches in PubMed, with the combination providing the largest number of results being the one selected for the expansion at each stage (for transparency, the individual searches and number of results received for each is available from Open Science Framework: https://osf.io/rk2cn/).

After each expansion, title and abstract review was performed for the combination that received the most results. This process of identifying the optimal combination of search terms, expanding the search string, and performing title and abstract review was continued until the number of new publications eligible on abstract review was approximately 1% of the total (see Table 1). The major assumption with this method was that if successive expansions yielded diminishing numbers of potentially eligible publications, and the most recent expansion yielded a relatively small addition to the pool, stopping the expansion at this point was unlikely to lead to a major loss of information. This search strategy has previously been described by Duffy *et al*,^11^ who found that 92% of articles were identified before reference list searches (60 of 65 articles were identified through the database searches alone).

To minimise the risk of excluding eligible studies not available on PubMed, the final search was repeated in PsychInfo (see Table 1) and the reference lists of selected publications were hand-searched by both reviewers. All searches were performed on 6 June 2021.

### Inclusion and exclusion criteria

Publications were included in the review if they:

- explored patients’ and/or PCPs’ attitudes or experiences with remote consultations in primary care;
- used a qualitative, quantitative, mixed-methods, or multi-methods research design; and
- were published after 2019 (that is, once the pandemic began) in a peer-reviewed journal.

Publications were excluded if they:

- were not available in English;
- were conducted before 2020 (that is, before research into the use of remote consultations during the COVID-19 pandemic began);
- were not published in a peer-reviewed journal; or
- did not involve either patients or PCPs.

### Screening procedure

All publications were assessed for eligibility by two reviewers. Each reviewer assigned articles a score of 1 (‘eligible’) or 0 (‘not eligible’), based on their title. Any article that received a combined score of 1 (considered eligible by one reviewer) or 2 (considered eligible by both reviewers) underwent abstract review. As with title review, each article that underwent abstract review was assigned a score of 1 or 0 by both reviewers, and underwent full article review if they received a combined score of 1 or 2. Unlike title and abstract review, articles that underwent full article review were only accepted for inclusion if they received a score of 2 (that is, considered eligible by both reviewers). Articles that received a score of 1 underwent discussion between the two reviewers until a decision was made, while articles that received a score of 0 were not discussed and were excluded from the review.

### Data extraction

Qualitative and quantitative data regarding the experiences of PCPs and patients were extracted from selected articles by one reviewer, with a proportion (20%) checked by a second reviewer. Data on the author, year of publication, country of origin, population (that is, patients or PCPs), sample size, study design, and type of analysis were also extracted. All data were extracted using customised Excel templates (for transparency, the raw data extracted are available in Supplementary Tables S3, S4, and Open Science Framework: https://osf.io/rk2cn/).

### Data analysis

Qualitative data on the experiences of PCPs and patients were analysed (separately) using thematic synthesis.^12^ In the first instance, two authors coded a proportion (*n* = 4, 33%) of the studies using line-by-line coding. A coding framework was subsequently developed and applied to the remaining transcripts (*n* = 8, 67%) by one of the authors (PV). Several further codes were subsequently added to the framework as new transcripts were analysed (previously coded transcripts were then revisited to check for the presence of newly identified codes). Finally, superordinate themes, themes, and subthemes were developed by both authors through an iterative process of comparing, re-examining, grouping, and re-grouping the codes until consensus was achieved. The data were coded and analysed in Excel; the number of studies in which subthemes were identified was also reported, to help assess the extent to which they might be important.

Quantitative data were analysed using narrative synthesis. One author extracted the quantitative data from the relevant studies and grouped them according to the subthemes derived from the thematic synthesis. An iterative process of grouping and re-grouping the quantitative data into subthemes was then performed by both authors until agreement was achieved. Quantitative data that could not be grouped under an existing subtheme were reviewed and discussed by both reviews; new specific subthemes were then developed for these codes and added to the framework.

### Rigour

Interpretive validity was achieved through the inclusion of a second reviewer, who confirmed that relevant data were extracted by the first reviewer. Similarly, reliability of data interpretation (that is, theoretical validity) was maintained through the inclusion of a second reviewer (RK) who reviewed and discussed interpretations with the first reviewer (PV).

### Transparency

For transparency, this review was registered with PROSPERO (Reference: CRD42021256566), was written in accordance with PRISMA guidelines (see https://osf.io/rk2cn/), and the data made publicly available on Open Science Framework: https://osf.io/rk2cn/.

## Results

### Database and reference list searches

**Figure 1.**
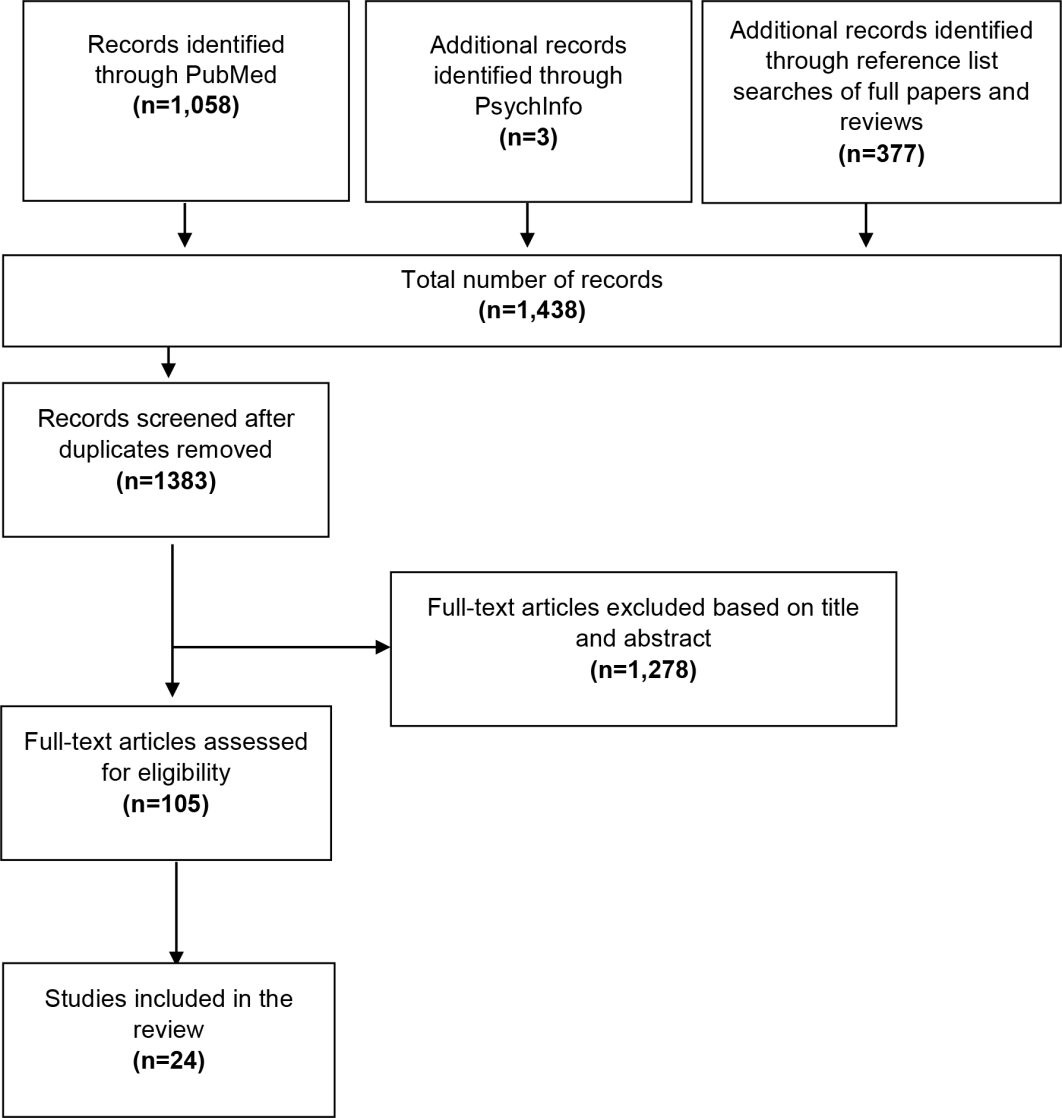
PRISMA flow diagram

In total, 1438 articles were identified through the database and reference list searches. After removing duplicates (*n* = 55), 1383 articles were eligible for title and abstract review, of which 105 passed and underwent full article review. A total of 24 articles were subsequently deemed eligible and were included in the review. An overview of the selection process is provided in Figure 1.

### Study characteristics

Table 2 presents a summary of the studies included in the review (a detailed overview of each study is provided in Supplementary Table S2). The majority of studies were performed in either the US (*n* = 6, 25%)^13–18^ or Europe (*n* = 7, 29%),^19–25^ used a quantitative design (*n* = 12, 50%),^14–18,25–31^ and explored the experiences of PCPs only (*n* = 16, 67%).^13,15,17,19,20,22–29,31–33^

**Table 2.** Summary of characteristics of articles included in the review

Design feature	Studies, *n* (%)
**Setting**	
US	6 (25%)^13–18^
Australia	4 (17%)^27,28,35,36^
UK	4 (17%)^21,22,24,25^
Belgium	2 (8%)^19,20^
Germany	1 (4%)^23^
Iran	1 (4%)^30^
Italy	1 (4%)^26^
New Zealand	1 (4%)^34^
Norway	1 (4%)^29^
Oman	1 (4%)^32^
Romania	1 (4%)^31^
Not specified	1 (4%)^33^
**Population**	
PCPs	16 (67%)^13,15,17,19,20,22–29,31–33^
Patients	5 (20%)^16,21,30,34,36^
PCPs and patients	3 (13%)^14,18,35^
**Sample size**	
10–20	3 (13%)^13,15,20^
21–100	7 (29%)^14,21–35,32,35^
101–500	7 (29%)^19,25,26,28,30,31,33^
>500	7 (29%)^16–27,29,34,36,36^
**Study design**	
Quantitative	12 (50%)^14–18,25–31^
Qualitative	8 (33%)^13,19–32,35,32,35^
Mixed-methods	2 (8%)^33,34^
Multi-method	2 (8%)^23,36^

PCP = primary care physician.

### Data analysis (qualitative and quantitative results)

**Figure 2.**
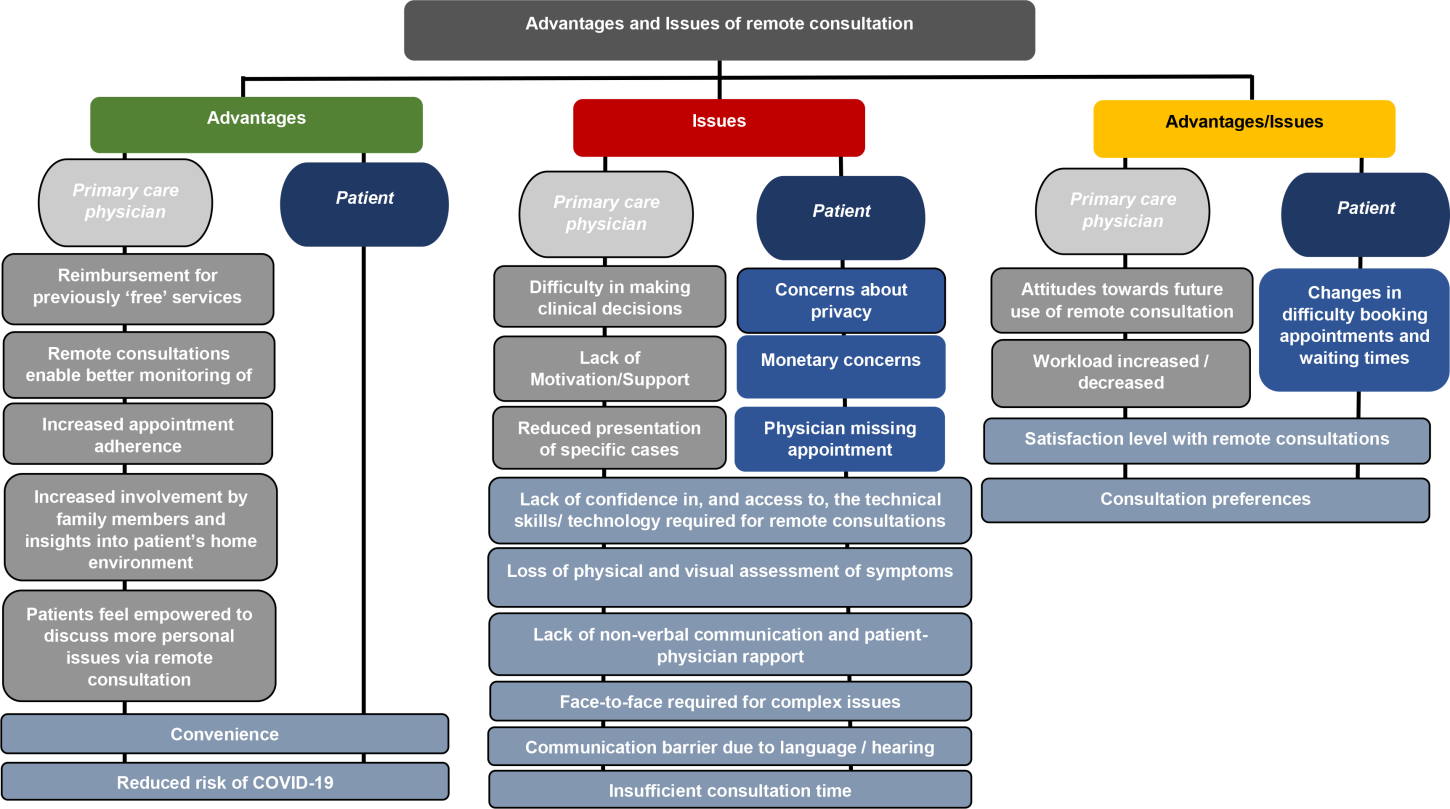
Perceived advantages and issues with remote consultations during the COVID-19 pandemic

Two major themes were identified within the data, namely the ‘perceived advantages’ and ‘perceived issues’ with remote consultations. These were further differentiated into those perceived by PCPs, those perceived by patients, and those perceived by both (see Figure 2 for an overview of the coding framework, and Table 3 for a comprehensive list of the number of studies for which each advantage and issue was identified). In some cases, one population perceived advantages or issues with remote consultations, that they believed affected the other population, but not themselves (for example, PCPs perceived advantages and issues with remote consultations which they believed affected patients but not PCPs). As these statements were not verified (that is, not reported by the group they were reported to affect), they were coded according to the group that reported them, and the group to which the advantage or issue related was specified (for example, if it was the PCP who stated that a specific quality of remote consultations was an advantage or issue for patients, and there were no data to support this from patients themselves, then this was coded as a PCP perception, not a patient perception, and specified in the label for the subtheme). There were no cases where advantages and issues were perceived by both groups (that is, patients and physicians) and were said to affect only one group (for example, patients). There were, however, cases where specific advantages and issues were perceived by both and reported to affect both. These were simply coded as 'both'.

**Table 3.** Quantification of advantages and issues

Advantages and issues	Articles (telephoneconsultations), *n*	Articles (videoconsultations), *n*	Articles (both video andtelephone consultations), *n*	Total articles, *n*
**Advantages**				
**Advantages (PCPs)**				
Reimbursement for previously ‘free’ services	1	–	4	5^15,17,18,25,27^
Remote consultations enable better monitoring of cases	3	–	1	4^13,20,22,31^
Increased appointment adherence	1	–	1	2^13,24^
Increased involvement by family members and insights into patient’s home environment	–	–	1	1^13^
Patients feel empowered to discuss more personal issues via remote consultation	1	–	–	1^13^
**Advantages (patients and PCPs)**				
Convenience	9	2	–	11^13,16,18,21–25,31,33–35–35^
Reduced risk of COVID-19	3	–	–	3^19,32,35^
**Issues**				
**Issues (PCPs)**				
Difficulty in making clinical decisions	3	1		4^19,20,29,31^
Lack of motivation or support	–	–	3	3^18,22,25^
Changes to consultation type	2	–	–	2^19,28^
**Issues (patients)**				
Concerns about privacy	3	–	1	4^16,30,33,34^
Monetary concerns	3	–	–	3^16,34,35^
Physician missing appointments	1	–	–	1^21^
**Issues (patients and PCPs)**				
Lack of confidence in, and access to, the technical skills or technology required for remote consultations	8	1	6	15^13,15–22,24,25,31–36,31–36^
Loss of non-verbal communication and patient–physician rapport	6	2	1	9^13,19,21,22,25,31,33–35^
Loss of physical and visual assessment of symptoms	5	2	1	8^13,19,21,22,25,29,33,34^
Face-to-face required for complex issues	6	3	–	9^13,16–21,22,29,32,33,33^
Communication barrier owing to language or hearing difficulties	4	–	1	5^13,17,25,32,34^
Insufficient consultation time	1	1	1	3^13,16,33^
**Advantages/issues**				
**Advantages/issues (PCPs)**				
Attitudes towards future use of remote consultation	3	1	2	6^13,18,22,25,29,31^
Workload increased or decreased	5	1	–	6^13,19,20,22,32,33^
**Advantages/issues (patients)**				
Changes in difficulty booking appointments and waiting times	3	–	–	3^25,34,35^
**Advantages/issues (patients and PCPs)**				
Satisfaction level with remote consultation	5	2	1	8^14,16,18,21,29–33,33^
Consultation preferences	–	3	2	5^16,18,25,29,33^

PCP = primary care physician.

The following provides a description of each of the advantages and issues identified (examples of quantitative results and patient and PCP quotes can be found in Supplemetary Tables S5A and S5B, respectively).

### Perceived advantages with remote consultations for PCPs (only)

#### Reimbursement for previously ‘free’ services

Several studies (*n* = 5) found that reimbursement of remote consultations during the COVID-19 pandemic was an advantage reported by many PCPs, who indicated that they had previously provided these services for free.^15,17,18,25,27^ PCPs endorsing this also indicated that should they be required to continue delivering them at this level in the future, it would be important for governments and insurance providers to continue reimbursing them for these consultations.

#### Remote consultations enable better monitoring of cases

Several articles (*n* = 4) also found that PCPs felt remote consultations enabled better monitoring of cases, as patients suffering from a chronic condition, such as diabetes, could quickly and easily adjust their medication over the phone, without having to wait for a face-to-face appointment to become available.^13,20,22,31^

#### Increased appointment adherence

Another key advantage of remote consultations reported in some articles (*n* = 2), was that they often resulted in higher appointment adherence compared with face-to-face consultations, with patients missing fewer telephone and video appointments.^13,24^

#### Increased involvement by family members and insights into patient’s home environment

In one article, PCPs reported that another advantage of remote consultation was that it enabled them to see their patient's home environment, giving them insights into patients' living conditions.^13^

#### Patients feel empowered to discuss more personal issues via remote consultation

In one study, PCPs commented that, in some cases, telephone consultations helped patients to articulate their symptoms more clearly, and that face-to-face consultations could sometimes be intimidating, especially when patients are sharing sensitive information.^13^

### Advantages with remote consultations for both PCPs and patients

#### Convenience

Several studies reported that PCPs and patients found remote consultations to be more convenient than face-to-face appointments.^13,16,18,21–25,31,33–35–35^ For example, in one study, 47% of patients indicated that they saved >30 minutes with remote consultations compared with face-to-face.^18^

#### Reduced risk of COVID-19

Reduced risk of infection to PCPs and patients was cited as an advantage of remote consultations in three studies.^19,32,35^ For example, in one study, a physician commented that patients prioritised reducing infection risk, especially patients who were suffering from chronic conditions.^32^

### Issues with remote consultations for PCPs (only)

#### Difficulty in making clinical decisions

Several articles (*n* = 4) reported that PCPs sometimes found making clinical decisions was more difficult when conducting remote consultations.^19,20,29,31^ A quantitative article, for example, found that 64% of PCPs felt that remote consultation negatively affected their judgment in making decisions.^31^ In the qualitative literature, PCPs explained that difficulty making clinical decisions is exacerbated in remote consultation, because they heavily depend on the patient’s ability to communicate their symptoms (as they are unable to see or feel them).^19^

#### Lack of motivation or support from policy regulators

In some studies (*n* = 3), PCPs reported that they were required to implement remote consultations, and received little to no support to do so.^18,22,25^ In addition, two studies found that PCPs felt that there was a lack of guidelines and system infrastructure.^18,25^ In one study, 67% of PCPs felt there was a lack of appropriate Current Procedural Terminology codes for the documentation of remote consultation visits.^18^

#### Reduced presentation of specific cases

Two articles found that PCPs were encountering fewer patients for specific types of illness.^19,28^ In one study, a PCP reported they were seeing fewer patients with chronic illnesses such as diabetes, and stated that these were being managed less effectively than in the past.^19^

### Issues with remote consultations for patients (only)

#### Concerns about privacy

Several quantitative and mixed-methods studies (*n* = 4) found that patients expressed concerns regarding the security and privacy of their conversation with the physician.^16,30,33,34^ In the qualitative literature, patients explained that concerns mainly related to the fact that many telemedicine visits took place in the home environment, where family members might overhear.^34^

#### Monetary concerns

Several studies (*n* = 3), found that patients had reservations about whether telemedicine consultations, which tend to be shorter, should be charged the same as face-to-face visits.^16,34,35^ Moreover, two studies found that patients pointed out that telemedicine visits, which required subsequent face-to-face visits, were charged twice, increasing the total cost.^34,35^

#### Physician missing appointment

One qualitative study found that caregivers of patients discussed issues with telephone consultations, and stated that *‘doctors did not always keep appointments, and either phoned at different times than agreed or did not call at all’*.^21^ For caregivers who do not live with the patients, this was reported to be a significant inconvenience.^21^

### Issues with remote consultations for both PCPs and patients

#### Lack of confidence in, and access to, the technical skills or technology required for remote consultations

Lack of confidence in, and access to, the technical skills or technology required for remote consultations was the single most frequently coded issue identified in the extant literature (*n* = 15).^13,15–22,24,25,31–36,31–36^ In the quantitative literature, one study found that >90% of patients reported that they had the necessary technology for remote consultation in the US, of which 20% reported that they had difficulty connecting to it.^18^ In a separate study, 26% of patients felt that using teleconsultation was too complicated,^36^ and, in another study 30% expressed that they had technical issues before or during the visit.^16^

In the qualitative literature, these technical challenges were mainly observed in older adults. Both patients and PCPs acknowledged that using a computer was generally more difficult for older adults.^13,21,22,24,32,34–36^

In addition to technological knowledge, several studies (*n* = 9) found that PCPs also expressed that the lack of access to technology was an issue — especially for patients living in rural areas — including poor connection, bad reception, poor audio quality, and video calls dropping out.^13,21,22,24,32–36^ A quantitative study, conducted in the US confirmed this finding, reporting that 12% expressed issues related to sound, 35% had video issues, and 40% were not able to connect.^16^

Two quantitative articles also stated that 25–50% of patients have difficulty using their device and/or video telehealth platform.^18,36^ Moreover, PCPs in qualitative literature had various technology problems, such as lack of mobile phones, highlighting the importance of technology while conducting remote consultation.^13,21,22,24,32–36^

#### Loss of physical and visual assessment of symptoms

Loss of physical and visual assessment of symptoms was reported to be an issue in eight studies.^13,19,21,22,25,29,33,34^ A quantitative article found that 38% of PCPs stated the inability to conduct a physical examination to the degree required, and that 16% of PCPs felt that assessing physical health status was a challenge (4% could not hear them properly during a video consultation visit).^25^ PCPs in a qualitative study pointed out that skin rashes were extremely difficult to treat, owing to patient’s inability to explain them.^13^ The qualitative literature suggests these issues appear to be related to emerging or growing physical problems, which are more difficult to communicate over the phone.

#### Loss of non-verbal communication and patient–physician rapport

In several studies (*n* = 9),^13,19,21,22,25,31,33–35^ PCPs stated that the loss of non-verbal communication was an issue, reporting that some patients found it extremely difficult to express themselves during remote consultation. Patients also felt that telemedicine was impersonal, even if they knew the doctor.^34^

Finally, three studies found that PCPs and patients felt disconnected, owing to the loss of physical touch during the remote consultation.^19,25,31^ They believed it to be a significant part of the patient–physician relationship and building this bond with a new patient became even more difficult.^19^

#### Face-to-face required for complex issues

Face-to-face was usually required for complex issues and was a challenge reported in multiple articles exploring physician and patient perspectives (*n* = 9).^13,16–21,22,29,32,33,33^ The quantitative literature helped quantify the extent of the problem. One article reported that only 50% of PCPs felt remote consultations were suitable for the overall visit they had conducted,^29^ while another reported that 90% of patients in the US did not recover from their illness by consulting remotely, and had to visit an urgent centre or were sent to medical centres for evaluation.^16^

Results were similar for patients and their caregivers, who said they prefer face-to-face consultations over telephone consultations, as it gives them more confidence to express their symptoms clearly.^21^

#### Communication barrier owing to language or hearing difficulties

Communication was an issue observed by both PCPs and patients in several studies (*n* = 5).^13,17,25,32,34^ PCPs reported that some of the patients could not communicate as they did not speak the same language.^13,32^ The effect of language barrier differed for PCPs according to the social vulnerability of the area they served. One quantitative article stated that providers in high-social vulnerability index areas were twice as likely to report language barriers as a concern.^17^ In addition to language, one study found that patients with hearing difficulties also found remote consultations difficult.^34^

#### Insufficient consultation time

In the quantitative literature, one study found that a small number of patients (7%) felt that the amount of time allocated to remote consultation was not sufficient.^16^ In the qualitative literature, similar observations were made by PCPs. In one study, a physician explained that the reason why consultations were shorter was that there was less talking being done by the patient,^33^ while another one felt patients wanted to spend less time on the phone.^13^

### Advantages/issues with remote consultations for PCPs (only)

#### Attitudes towards future use of remote consultation

Two quantitative articles stated that 85–95% of PCPs believed that remote consultation should be continued in the future.^26,31^ However, an article from Norway stated that only 49% of PCPs were motivated to continue video consultations post-COVID-19.^29^ Two studies (conversely) found that PCPs were worried that remote consultation could lead to PCPs’ provision of on-demand care and potential burnout.^13,22^

#### Workload increased or decreased

There were mixed views about the impact of remote consultations on workload for PCPs reported across the literature (*n* = 6).^13,19,20,22,32,33^ Two studies found that PCPs were more structured in their working schedule, dividing their work between phone triage, consultation, and face-to-face consultation.^20,22^ Conversely, other studies found that PCPs found splitting their day to be highly stressful.^19,32^ For example, one article stated that PCPs found balancing in-person and remote consultation schedules a bit hectic, which often resulted in PCPs running late for their visits.^13^

### Advantages/issues with remote consultations for patients (only)

#### Changes in difficulty booking appointments and waiting times

A quantitative study found that 42% of patients raised concerns regarding the unavailability of physician appointments in a time of need.^36^ These findings were partially echoed in the qualitative literature.^35^ One study had a contrasting finding that patients reported ease in getting a remote consultation.^34^

### Advantages/issues with remote consultations for PCPs and patients

#### Satisfaction level with remote consultations

Across studies (*n* = 8), a mixed reaction was observed concerning the satisfaction level with remote consultation.^14,16,18,21,29–33,33^ In Norway, 85% of the PCPs perceived that their patients were satisfied with video consultations.^29^ However, in a study conducted in the US, 83% of PCPs believed they could not provide adequate care by only using virtual visits, and only 57% of PCPs were satisfied with the interpersonal patient connection established during telephone consultation.^18^ These findings were echoed in the qualitative and mixed-methods literature.^21,33^

#### Consultation preferences

Across the literature (*n* = 5), PCPs and patients had diverse preferences regarding their primary care consultation.^16,18,25,29,33^ 96% of PCPs based in the US indicated that they would like to continue virtual visits in the future, of whom 64% preferred video consultation, and only 9% preferred telephone consultation.^18^ In a separate study, 61% of PCPs specified that video consultation was better suited for follow-up than new disease.^29^

## Discussion

### Summary

This review identified a range of advantages and issues with using remote consultations in primary care during the COVID-19 pandemic. Some of the key advantages described by patients and PCPs were that remote consultations are more convenient than face-to-face appointments, and reduce the risk of patients and staff getting COVID-19. Some of the key issues included a lack of confidence in, and access to, adequate technology, and the loss of non-verbal communication between patients and PCPs.

This review identified a number of contradictions within the literature, with several aspects of remote consultations being discussed as both advantages and issues. For example, some PCPs stated that their workload had increased, while others reported that it had decreased. Such contradictions are likely owing to differences in the settings in which the studies were performed, and the extent to which they were affected by COVID-19. For example, one country in which workload was said to increase was the US,^13^ which modelling suggests was to be one of the most affected countries in terms of cases ( thus, more patients may have been calling practices about their symptoms).^37^

Several disparities in the perceived advantages and issues with using remote consultations in primary care were also observed between patients and PCPs. For example, patients often reported that remote consultations were too short, and that they did not always have time to discuss everything they wanted to.^21^ By comparison, PCPs felt that the shorter duration of remote consultations enabled them to manage their time better, and believed that patients came to the point more quickly without indulging in ‘*small talk*’.^13^

### Strengths and limitations

This review has several strengths. First, it included both quantitative and qualitative research. Second, two reviewers reviewed the titles and abstracts of potentially eligible articles, minimising the chances that relevant articles were erroneously excluded. Finally, two reviewers coded and analysed the data, improving the reliability the findings.

This review also has several limitations. First, only two databases were searched. Second, only studies written in English were included. Finally, no formal quality assessment was conducted, and the results were taken at face value.

### Comparison with existing literature

The results of this review are similar to those published in a previous systematic review conducted by Mold *et al* before the pandemic,^38^ which assessed the literature exploring patient and physician attitudes towards delivering primary care via electronic consultation, email, messaging, and video links. Their review similarly found that patients had concerns about the privacy and security of e-consultations.^38^ The present review, however, made several unique observations; for example, in addition to privacy concerns, there were monetary concerns, and issues with PCPs missing appointments, which were expressed by patients. One potential explanation for these differences between the two reviews is that one focused on e-consultations, while the other focused on telephone and video consultations.

### Implications for research and practice

This review has several implications for policy and future research. First, it suggests that patients and PCPs are generally satisfied with remote consultations, and believe them to be preferable for specific appointments, such as follow-up of a previous face-to-face appointment. Second, it suggests that further research is required in countries with healthcare system models unrepresented, or underrepresented, in the present review. Finally, this review suggests several situations where remote consultations should not be used, such as where the patient is presenting to the GP for the first time, or in relation to a new condition or symptom.

## Provenances

Freely submitted; externally peer reviewed.

## Data

The data have been made publicly available on Open Science Framework: https://osf.io/rk2cn/.

## Trial registration number

This review was registered with PROSPERO (Reference: CRD42021256566), and was written in accordance with PRISMA guidelines (https://osf.io/rk2cn/).
